# Dual in Utero Electroporation in Mice to Manipulate Two Specific Neuronal Populations in the Developing Cortex

**DOI:** 10.3389/fbioe.2021.814638

**Published:** 2022-01-12

**Authors:** Longbo Zhang, Stephanie A. Getz, Angelique Bordey

**Affiliations:** ^1^ Departments of Neurosurgery, And National Clinical Research Center of Geriatric Disorders, Xiangya Hospital, Central South University, Changsha, China; ^2^ Departments of Neurosurgery, And Cellular and Molecular Physiology, School of Medicine, Yale University, New Haven, CT, United States

**Keywords:** *in utero* electroporation, neuron, development, cortex, neuroonal connectivity

## Abstract

Precise regulation of gene expression during development in cortical neurons is essential for the establishment and maintenance of neuronal connectivity and higher-order cognition. Dual in utero electroporation provides a precise and effective tool to label and manipulate gene expression in multiple neuronal populations within a circuit in a spatially and temporally regulated manner. In addition, this technique allows for morphophysiological investigations into neuronal development and connectivity following cell-specific gene manipulations. Here, we detail the dual *in utero* electroporation protocol.

**GRAPHICAL ABSTRACT:**
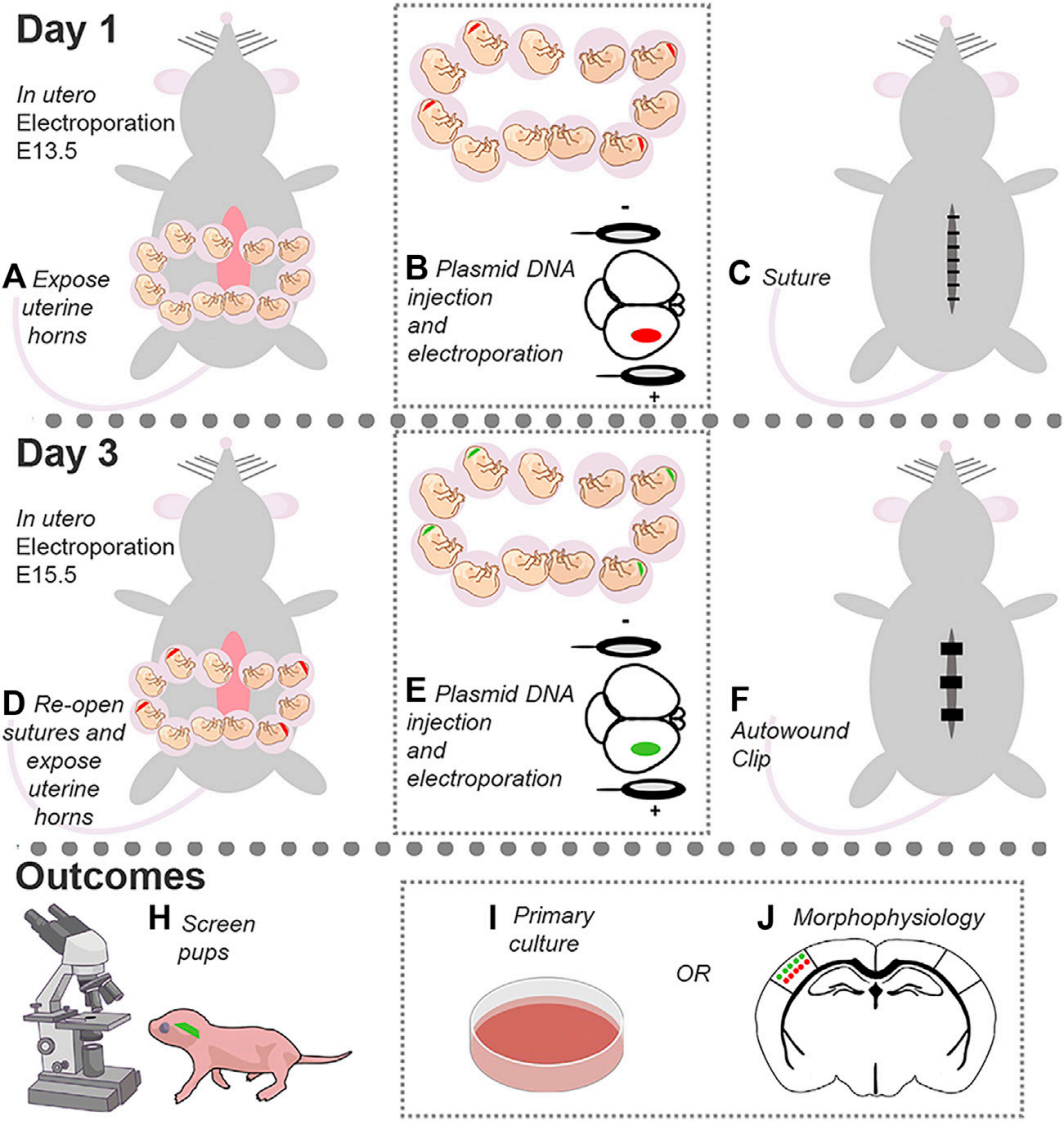


## Introduction

**FIGURE 1:**
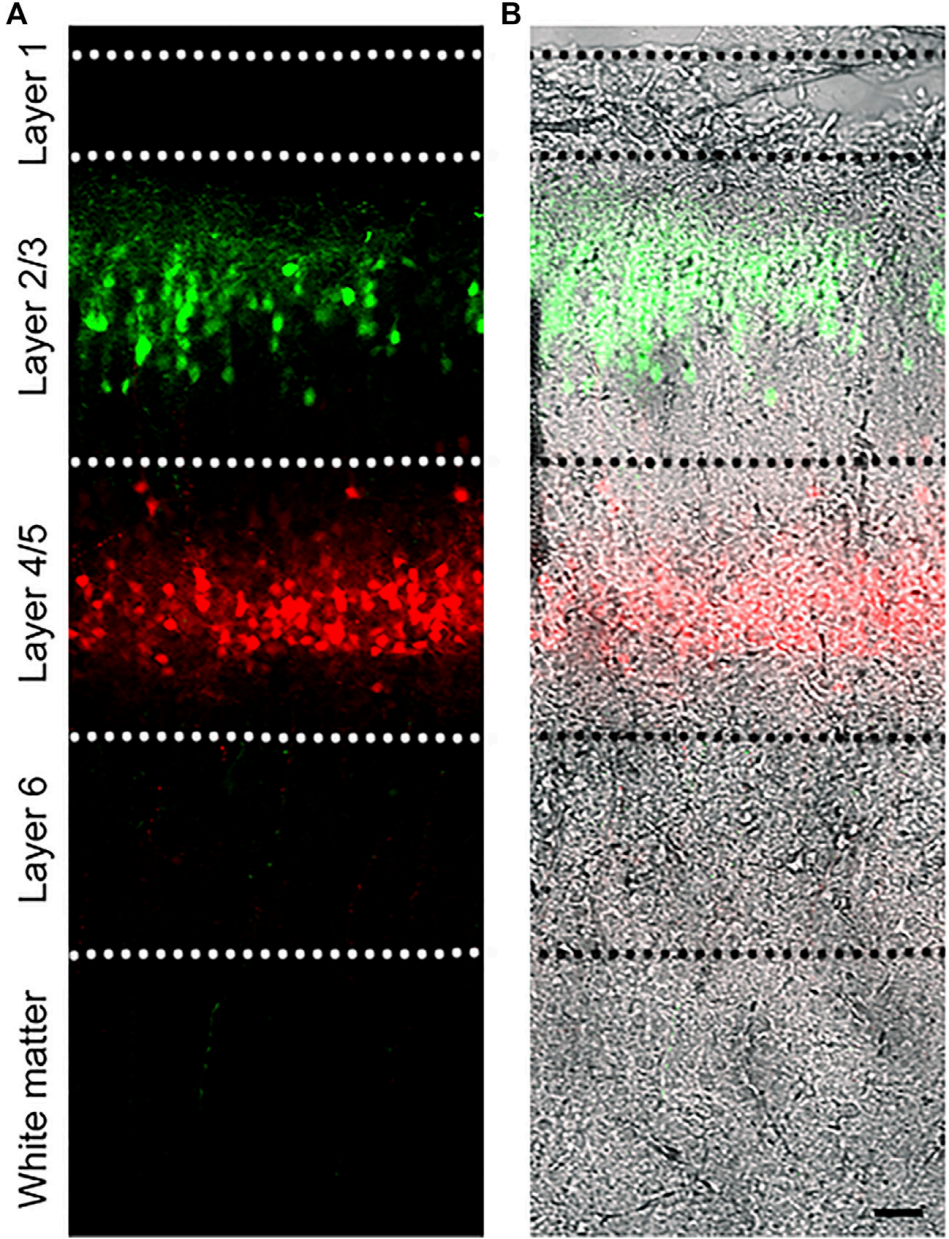
Dual IUE image in the SSC. **(A)**: Image of a postnatal day (P) 14 mouse coronal section containing GFP-expressing L2/3 cortical neurons and tdTomato-expressing L4/5 neurons following IUE at E15.5 and E13.5, respectively. **(B)**: Fluorescent image overlaid with bright field. Bar: 50 µm.

In utero electroporation (IUE) is a powerful tool to target specific neuronal populations in the developing cortex with temporal and spatial resolution (Saito and Nakatsuji, 2001; Fukuchi-Shimogori and Grove, 2001). The protocol below describes a detailed protocol to perform dual IUE to target layers (L) 2/3 and 4/5 pyramidal neurons in the somatosensory cortex (SSC) with different fluorescent markers to study neuronal development, including dendrite growth and synaptic connectivity (Zhang et al., 2021) (Figure 1). To do this, DNA in a plasmid form is introduced into the lateral ventricle of embryos, and an electric pulse is applied to drive the DNA into the desired neural progenitor cell populations. IUE at embryonic day (E) 13.5 and 15.5 is targeted to neural progenitor cells that generate pyramidal neurons destined to reach L4/5 and L2/3, respectively (Molyneaux et al., 2007). Mechanistically, the current from the tweezertrode anode drives the negatively charged DNA toward the desired cortical region, while the electrical pulses generate pores to facilitate DNA delivery into the selected progenitor cell population. The DNA is then passed on to the daughter cells, i.e., neurons, from the progenitor cells upon cell division. These newborn neurons migrate to their target cortical layers and then differentiate into mature pyramidal neurons. Thus, a single IUE allows to manipulate a specific neuronal population within a discrete cortical location.

This protocol can be utilized to target different cortical regions, such as the medial prefrontal cortex (mPFC), as well as the hippocampus. In the DNA-encoding vector, different promoters can be used to restrict gene expression in neurons while bypassing expression in neural progenitor cells (e.g., doublecortin promoter; (Lin et al., 2016). This allows to selectively examine the impact of gene manipulations in neurons vs neural progenitor cells. Overexpression and short hairpin RNA-encoding vectors as well as CRISPR/Cas9 system can be used to examine the impact of gene overexpression, knockdown, or mutations on neuron development. Finally, the IUE procedure can be adapted to target unilateral or bilateral cortices. Taken together, dual IUE provides an excellent tool to target and manipulate one or multiple cell populations in a temporally- and spatially regulated manner to study neuronal development and ultimately connectivity, network activity, and animal behavior. Cortices can also be collected and plated for primary neuronal culture studies.

**Key Resources Table:** 

Reagent or Resource	Source	Identifier
Chemicals, peptides, and recombinant proteins		
Fast Green FCF	Sigma	Ref F7252
PBS, 1x, pH 7.4	Quality Biological	Ref 114-058-101
Paraformaldehyde 32% Solution	Electron Microscopy Sciences	Ref 15714-S
Experimental models: Organisms/strains		
CD-1 IGS Mice	Charles River Laboratories	Ref 022
Recombinant DNA		
pCAG-GFP	Addgene	Addgene # 11150; Matsuda et al. (2004) PNAS
pCAG-tdTomato	Addgene	Addgene # 83029; Pathania et al. (2012) PLos One
pCALNL-DsRed	Addgene	Addgene # 13769; Matsuda et al. (2007) PNAS
pCAG-Cre	Addgene	Plasmid # 13775; Matsuda et al. (2007) PNAS
Surgical tools		
Small Round-Tipped Forceps; 3 mm O.D., 2.2 mm I.D.	Hammacher Instrumente	Ref HSC_703–93
Large Round-Tipped Forceps; 6 mm O.D., 4.8 mm I.D.	Hammacher Instrumente	Ref HSC_703–96
Microdissecting Scissors; Straight; 23 mm Blade Length	ROBOZ; SouthPointe Surgical Supply	Ref RS-5910SC
Webster Needle Holder; Serrated, Extra Delicate	WPI	Ref 14109
5–0 UNIFY PGA Absorbable Braided, Coated Suture, Undyed; 18″	AD Surgical	Ref S-G518R13-U
Autoclip 9 mm	Clay Adams Brand	Ref 300216
MikRon 9 mm AutoClip Applier	Clay Adams Brand	Ref NC9021392
Anesthesia system		
Vaporizor; EZ-7000 Classic System	Vaporizor; EZ-7000 Classic System	Vaporizor; EZ-7000 Classic System
Isoflurane; 100 ml	Isoflurane; 100 ml	Isoflurane; 100 ml
IUE tools		
Electrode Puller	Narishige Japan	Model PP-830
Borosilicate Glass with Filament; Fire Polished; O.D.: 1.5mm, I.D. 1.10 mm, 10 cm	Sutter Instrument	Ref BF150-110-10
Electro Square Porator; ECM 830	BTX Harvard Apparatus	Ref W3 45-0052
Tweezertrodes Platinum Plated 3 mm	BTX Harvard Apparatus	Ref 45-0487
Tweezertrodes Platinum Plated 5 mm	BTX Harvard Apparatus	Ref 45-0489
Tweezertrodes Cable Adaptors	BTX Harvard Apparatus	Ref 45-0204
15″ Aspirator Tube Assembly	Drummond Scientific	Ref 2-000-000
Pre- and post-surgery treatment drugs		
Rimadyl (carprofen); Injectable 50 mg/ml; 20 ml	Zoetis; Covetrus	Ref 024 751
Buprenorphine HCl Injection: 0.3 mg/ml; C3	Covetrus	Ref 059 122
lidocaine 2.5% and prilocaine 2.5% Cream	Akorn; McKesson	Cat. No. 1331487
Puralube Vet Ointment	Dechra; Medvet	Cat. No. PH-PURALUBE-VET
Screening and images collection		
Fluorescence Microscope	Olympus	Ref SZX16
FV1000	Olympus	
Vibratome	Leica	Ref VT1000S
Perfusion and fixation		
Forceps for Perfusion	ROBOZ	RS-5136
Large scissors for Perfusion	WPI	Ref 19,520
Medium scissors for Perfusion	WPI	Ref 191 210
Spring scissors for Perfusion	WPI	Ref 501 235
Micro spatula for Perfusion	Sigma	Ref Z513377
Other		
0.9% Sodium Chloride Injection; 10 ml	Hospira	Ref NDC 0409-4888-02
Sterile Empty Vial; 10 ml	Hospira	Ref 5816–11
30G x ½ (0.3 mm × 13 mm) PrecisionGlide Needle	Betcon, Dickinson and Company	Ref 305106
1 ml syringe; Tuberculin Slip Tip	Betcon, Dickinson and Company	Ref 309569
Polylined Sterile Field; Non**-**Fenestrated	Busse Hospital Disposables	Ref 696
Wahl Lithium-Ion Vacuum Trimmer Kit with Adjustable Vacuum Intake	Walmart	Cat. No. 9870
Non-Woven Sponges; Non-Sterile	McKesson	Ref 94442000
Prevantics	McKesson	Ref B108000
Alcohol Prep Pad	McKesson	Ref 58–204
30 CC syringe; Luer Lock Tip; Sterile	McKesson	Ref 16-S40C
Millex-GV Duapore PVDF Membrane; 0.22 um Filter unit	Millipore Sigma	Ref SLGVR33RS
Acrodisc Syringe Filter; 0.2 um Supor Membrane Low Protein Binding	Pall Life Sciences	Ref 4602
Parafilm; 4 in x 125 ft	Millipore Sigma	Ref P7793
Halogen Light Source	AmScope	Ref HL250 AY
Snuggle Safe Heat Pad	Walmart	Cat. No. 596688379

**Key Resources Table:** 

DNA Dilution		
Reagent	Final concentration	Amount
Plasmid DNA (pCAG-tdTomato)	1.5 μg/μL	Depends on concentration
Plasmid DNA (pCAG-GFP)	1.5 μg/μL	Depends on concentration
Fast green 0.25%	0.025%	2.5 µL
PBS 1x	n/a	Up to 10 µL
Total	n/a	10 µL

**Note:** Make the day of surgeries and store at room temperature once made. One or more plasmids can be used per condition. Fast green concentration can be as low as 0.01%.

**Optional:** to label a subset of single neurons within the electroporated region, use a combination of plasmids pCALNL-DsRed and pCAG-Cre at 1000:1 ratio.

**Key Resources Table:** 

Electroporator Setup	
Electroporator parameters	Value
Pulse Voltage	36 mV (E13.5); 39 mV (E15.5)
Programmable Pulses	5
Pulse Width	50 ms
Space Between Pulses	950 ms

**Key Resources Table:** 

Anesthesia System Setup		
Anesthesia parameters	Isoflurane	LPM
Induction	4%	NA
Maintenance	2%	NA
Primary Flowmeter	NA	2.5
Secondary Flowmeter	NA	1.5

## Materials and Equipment

## Methods

### DNA Dilution Preparation

Timing: 15 min.

1. Obtain DNA plasmids, filtered sterile PBS, and Fast Green (0.25%) for electroporation.
  a. Use a plasmid encoding a fluorescent protein under the CAG promoter (i.e., pCAG-tdTomato or pCAG-GFP; CAG is a cytomegalovirus [CMV] early enhancer fused to modified chicken actin promoter) pCAG-tdTomato or pCAG-GFP, to label neurons and detect efficient electroporation at birth in live mice.

Optional: use an inducible CAG promoter encoding DsRed following a floxed stop cassette (pCAG-*LoxP*-stop-*LoxP*-DsRed, called pCALNL-DsRed, addgene: #13769) together with pCAG-GFP and low concentration of pCAG-Cre to label a subset of GFP-expressing neurons within the electroporated region (Zhang et al., 2021).

2. Combine reagents so that there is a total of 10 µL plasmid DNA per mouse. DNA dilution can range from 1 to 4 μg/ μL. The table below uses 1.5 μg/ μL as an example. Dilute fast green to a final concentration of 0.025%. PBS will be used to bring the final volume to 10 µL.
3. Store stock DNA plasmids at −20°C and working aliquots at 4°C.

CRITICAL: Do not vortex DNA and avoid freeze-thawing. Flick the tube and spin down before use.

CRITICAL: Plasmid DNA concentration must be high enough, ideally, no lower than 5 μg/ μL, to allow for adequate expression in cells. Further, avoid thick plasmid DNA; if the DNA plasmid consistency is too viscous, then it will be challenging to use for electroporation.

### Electrode Preparation

Timing: 15 min.

**FIGURE 2:**
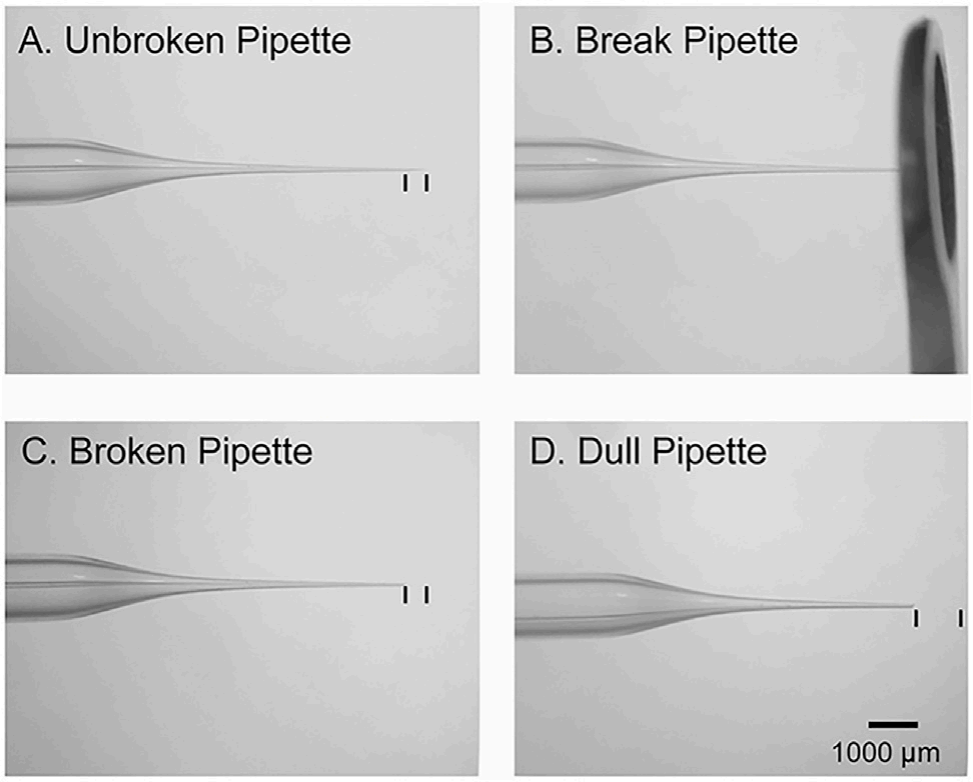
Micropipette preparation. **(A)**: Image of an intact glass micropipette that was generated by a vertical puller. The black lines denote the amount of tip that should be removed prior to insertion into the embryo. **(B)**: Image of the large round forceps used to break the tip of micropipette. **(C)**: Image of an adequately broken micropipette with an ∼45-degree-tip. The black lines denote the amount of tip that was removed. **(D)**: An example of a dull pipette. The black lines denote the amount of tip that was removed.

4. Pull electrodes using a glass micropipette puller.
  a. Have at least two electrodes per condition per animal when beginning surgeries.
  b. If using a dual-stage glass micropipette puller (Narashige Model PP-830), then heat electrodes to 70.5 °C for these experiments. Use only the bottom electrode.
5. Place the micropipette tip inside the 6 mm O.D. large round-tipped forceps and gently break off the tip to create an angle of about 45° (Figure 2). Break multiple sizes to test which tip diameter works best for each embryonic age.

CRITICAL: If the micropipette tip is too dull, then it will not penetrate the embryonic sac. If the tip is too thin, then it will be challenging to deposit plasmid into the brain. If there is clear damage to the cortex after electroporation, then the tip is likely too thick (Figure 2).

### Surgical Tool Preparation

Timing: 1.5 h.

6. Autoclave large and small round-tipped forceps, needle driver, and scissors. This can be completed the day of or the night before.
7. Place sterile 1x PBS into 30- or 50-ml syringes fitted with a 0.22 mm filter for surgeries.
8. Sterilize fenestrated drapes and gauze.

### Primary IUE

Timing: 0.5–1.0 h.

1. Prewarm the heating pad in a microwave and PBS to 37°C in a water bath. Wipe down the surgical area with 70% ethanol.
2. Just before beginning surgery, administer 0.1 mg/ kg body weight subcutaneous (s.c.) buprenorphine to timed-pregnant mice.
3. Anesthetize a E13.5 timed-pregnant mouse by placing it in the isoflurane induction chamber.
  a. Turn on oxygen, and open primary and secondary flowmeters. Set the secondary flowmeter to ∼1.5 LPM.
  b. Turn on isoflurane to 4% and wait until the mouse is immobile, then decrease the level to 2%.
4. After ∼3 min, take the mouse out of the chamber and shave the mouse’s belly with clippers while it is anesthetized.

Note: Suitable embryonic age can be differ depending on the regions.

CRITICAL: If the mouse starts waking up after completion of the shaving procedure, place it back in the induction chamber and repeat Step 3.

5. Move the mouse onto the heating pad with its abdomen exposed and place its nose into the nose cone.
  a. Put ophthalmic ointment on the eyes.
  b. Place 2.5% lidocaine prilocaine cream on its abdomen and tape the limbs down.
6. Wipe down the abdomen using alternative swabs of prevantics and alcohol. Do this three times, starting with prevantics and ending with alcohol.
7. Cover the abdomen with a piece of fenestrated sterile drape and gauze.
  a. Align the hole to be around the area of the incision.
  b. Drench the gauze with the warm, sterile PBS.
8. Pinch the skin using the small ringed forceps and lift skin, make a skin incision of ∼2 cm long though the midline with scissors.
9. Using the small ringed forceps, lift the muscle of abdominal wall and make an incision with scissors through the linea alba (white line) to avoid cutting of major blood vessels.

CRITICAL: Lifting the muscle before the incision of abdominal wall is important to avoid injuries of organs and embryos underneath. Begin with a small cut to allow the air in to push apart the abdominal wall and tissue underneath.

**FIGURE 3:**
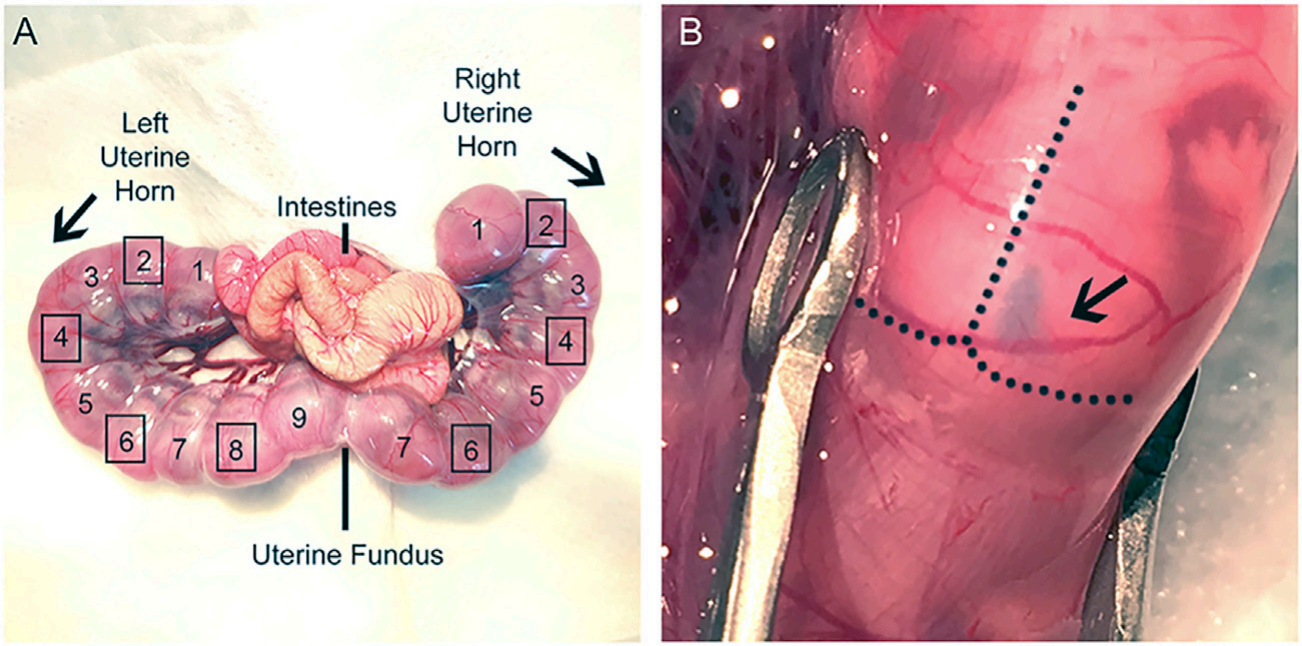
Uterine horns exposure during IUE surgery. **(A)**: Image of dissected uterine horns and labeling of desired IUE embryos. Uterine fundus is a useful marker to distinguish the two uterine horns and can be used as a reference to identify the electroporated embryos prior to the second IUE. Label embryos beginning at the uterine knots and continue sequentially to the uterine fundus for each uterine horn. Do not electroporate the embryos at the uterine knot or at the fundus (Left horn: 1, 9; Right horn: 1, 7). Squares surrounding the numbered embryos denote which embryos should receive IUE (Left horn: 2, 4, 6, 8; Right horn: 2, 4, 6). Electroporate embryos in an alternating fashion in the same hemisphere for both IUEs. **(B)**: Image of lateral ventricle injection. Plasmid DNA was injected into E15 embryo, and the right lateral ventricle was filled with fast green.

10. Gently move the uterine horns out through the incision using the large ring forceps and lay the uterine horns on the top of the gauze with the number of embryos counted and write the sequence of embryos in a notebook (Figure 3A). Regularly wet uterine horns with warm PBS.

CRITICAL: Carefully pull the uterine horns out of the abdominal cavity with the large ring forceps. Gently pull at the areas where embryos meet, rather than pulling directly on the embryos, to avoid damaging them. Alternatively, one can use fingers to gently move the embryos out while holding the peritoneum up and to the side with large ringed forceps.

11. Inject 1–2 μL plasmid DNA solution into the lateral ventricle using a mouth-controlled micropipette, which consists of a 1 ml syringe sans plunger, 15″ aspirator tube assembly, 0.22 mm filter, and micropipette (Figure 3B).

Note: The lateral ventricle should be filled with fast green if the injection is successful (Figure 3B).

CRITICAL: To increase survival rate after the second IUE, inject embryos in an alternating pattern.

CRITICAL: Avoid placing too much of the micropipette tip inside the embryonic sac. Doing so leads to holes, which can cause amniotic fluid to leak and increase the risk of abortion.

CRITICAL: Micropipette tips should not be reused for more than 10 embryos since the tip becomes blunt, which increases potential damage to embryos. Do not reuse electrodes across litters and conditions.

12. Place forceps-type electrodes (tweezertrode diameter: 2 mm) parallel to DNA-injected embryos (position and direction of electrodes depend on the desired target areas) and deliver 36 mV for 50 ms-long electric pulses with a 950 ms pulse interval.

Note: The electrodes should be placed in PBS-containing conical tube to keep them wet.

CRITICAL: The tweezertrode anode should be placed to direct the DNA toward the progenitor cell population of interest. For the SSC, the tweezertrodes will be orthogonally oriented with the anode on the barrel cortex. The embryo should move slightly when electrical pulses are being applied.

CRITICAL: Gently push the embryo towards the embryonic sac to ensure more efficient electroporation delivery. The limbs of embryo should move after each electric pulse.

13. Repeat steps 11–12 for the desired embryos. Keep track of which embryos and hemispheres are electroporated.

CRITICAL: Regularly drop warm PBS onto the uterine horns before moving to the next electroporation.

14. Reposition the horns carefully using the large ring forceps back into the abdominal cavity. Fill cavity with warm PBS.
15. Suture the abdominal wall and skin with absorbable PDS suture line.
16. Cover the skin incision with triple antibiotic.
17. Administer 5 mg/ kg/s.c. Rimadyl (carprofen).
18. Place the mouse in an empty cage on top of a heating pad until awake.
19. Upon waking, move the mouse back to its home cage. Put moistened food in the cage.

### Secondary IUE

Timing: 0.5–1.0 h.

20. Monitor mouse at 24 h post-surgery and administer 5 mg/kg/s.c. Rimadyl (carprofen).
21. 48 h after the first IUE (E15.5), and 10 min prior to surgery, administer 0.1 mg/kg/s.c. Buprenorphine.

**FIGURE 4:**
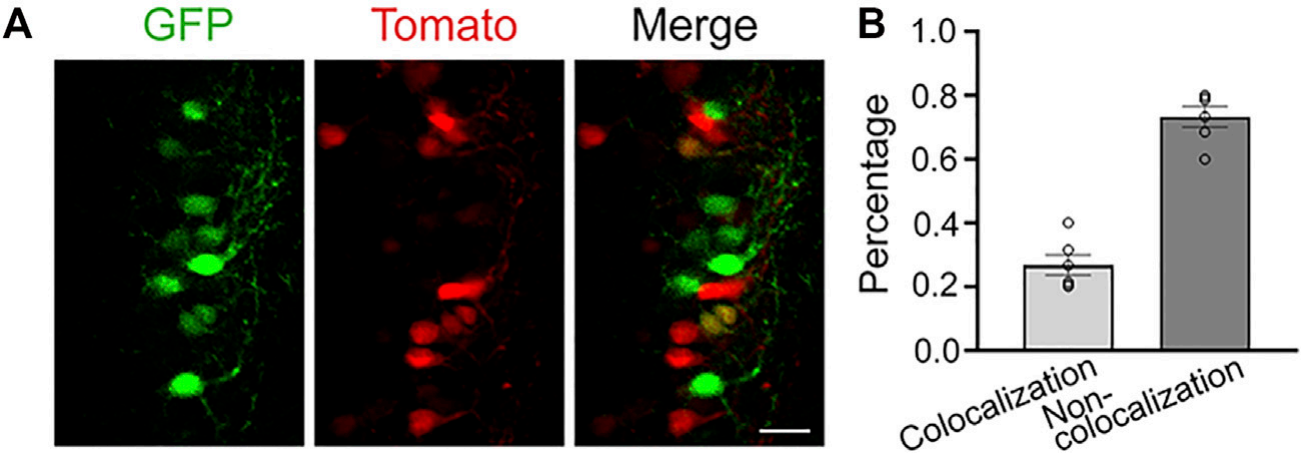
Dual IUE 12-h apart leads to colocalization between the two electroporated cell populations. **(A)**: Images of electroporated neurons in the SSC following a dual IUE 12-h apart (green: pCAG-GFP; red: pCAG-tdTomato). **(B)**: Quantification of the GFP and RFP colocalization and non-colocalization. Bar: 25 μm, 2 slices per mouse, N = 3 mice.

CRITICAL: Performing the second IUE too soon (e.g., 12 h instead of 48 h) following the first IUE results in a larger degree of co-localization between electroporated cell populations (Figure 4).

22. Anesthetize the mouse and repeat steps 1–7.
23. Using the small ringed forceps to pinch and lift skin, cut the suture line from the first surgery and gently open the original skin and abdominal wall incision.

Optional: Trim the skin and abdominal wall if needed.

24. Guide uterine horns out and lay them on the top of the gauze. Regularly wet uterine horns with warm PBS.
25. Find the electroporated embryos from last surgery and inject 1–2 μL plasmid DNA solution into the lateral ventricle using a micropipette. Make sure the lateral ventricle is filled with fast green.
26. Electroporate using forceps-type electrodes (Tweezertrodes diameter: 5 mm; parameter setting: 39 mV for 50 ms-long electric pulses, 950 ms pulse interval). Make sure the embryos move with the electrical pulses.
27. After finishing the second IUE of the embryos, reposition the horns carefully using the large ring forceps into the abdominal cavity. Fill cavity with warm PBS.
28. Suture the abdominal wall with absorbable PDS suture line. Next, place one suture in the middle of the incision on the skin and staple the skin closed using autowound clips.
29. Repeat steps 16–19.
30. Post-surgical care: monitor mouse every day for 72-h post-surgery and administer 5 mg/kg/s.c. Rimadyl (carprofen) daily.

## Expected Outcomes

Mice are typically born between E19 and E20. They can then be screened for fluorescence expression. IUE in CD1 mice are typically well-tolerated with 70–90% of electroporated pups being born. There could be some variability in the targeted region between mice and across litters. Practice can limit variability. In addition, including controls in the same litter is critical depending on the parameters (e.g., behavior) that are analyzed. Electroporated mice can be used for many purposes, including preparing fixed brain sections for imaging and morphological characterizations, preparing acute slices for electrophysiology, behavior analysis, and dissecting cells for *in vitro* experiments.

## Discussion

Dual IUE can target cortical neurons within the same cortical region but in different layers or target neurons in different cortical regions and layers. However, dual IUE may have a reduced efficacy compared to single IUE. One must be gentle with the embryos to maximize survival. When researchers are beginning this procedure, begin with a single IUE in the desired cortical location. Once it is established, then transition to dual IUE.

### Troubleshooting

#### Problem 1

Cannot penetrate the embryonic sac with the micropipette.

#### Potential Solution

The micropipette tip is too dull or wide. Change to a sharper tip and repeat. Proceeding with a micropipette tip that is too dull or wide could damage the embryo (Figure 2).

#### Problem 2

Cannot deposit the plasmid into the lateral ventricle.

#### Potential Solution

There could be multiple causes for this (Saito and Nakatsuji, 2001): The tip has bypassed or passed through the lateral ventricle and is somewhere else in the brain. Pull back on the tip while continuing to gently blow out the plasmid (Fukuchi-Shimogori and Grove, 2001). The micropipette tip is too thin to inject DNA solution into lateral ventricle. Change micropipette to a slightly larger tip diameter (Zhang et al., 2021). There is too much pressure on the pup. Loosen the grip on the pup and try again.

#### Problem 3

The embryonic sac is leaking amniotic fluid.

#### Potential Solution

The micropipette tip may be too dull or wide. Adjust micropipette sharpness or thickness accordingly (Fukuchi-Shimogori and Grove, 2001). The micropipette was placed too deep into the embryonic sac during plasmid injection. Ensure the micropipette is properly inserted in the lateral ventricle.

#### Problem 4

Popping of the embryonic sac during handling.

#### Potential Solution

If this happened, the embryos will not likely survive. Handle the embryos more gently.

#### Problem 5

The dam is bleeding.

#### Potential Solution

Although this procedure should not result in excessive bleeding, there could be blood between the skin and peritoneum. As long as it is only a small amount of blood, this will resolve as the surgery is completed. If an organ was ruptured, then there would be more blood within the peritoneal cavity. If so, euthanize the animal.

#### Problem 6

Few or no electroporated pups are born.

#### Potential Solution

The experimenter was likely not gentle enough with the embryos during surgery. The experimenter should put less pressure on the embryos, deposit the DNA plasmids with less force, and consider altering micropipette sizes. In addition, pups at the top of the uterine horn (near the knots) and at the junction between the two uterine horns should not be electroporated otherwise this increases the chances of spontaneous abortions.

#### Problem 7

Electroporated pups are born, but the fluorescent protein is expressed in subcortical or incorrect cortical locations.

#### Potential Solution

If the fluorescent reporter is in subcortical locations, then the micropipette was too deep. Ensure the plasmid DNA is properly injected into the lateral ventricle. If it is expressed in incorrect cortical locations, then adjust tweezertrode orientation to obtain the correct location.

#### Problem 8

Electroporated pups are born, but there is noticeable damage to the cortex or hydrocephalus.

#### Potential Solution

If there is damage to the cortex, then the micropipette was too dull. Hydrocephalus can happen with IUE, but it is uncommon. This could occur for several reasons: 1) too much pressure when depositing the plasmid into the lateral ventricle, 2) from the micropipette injection causing bleeding inside the lateral ventricle, or 3) inflammation. Ensure sterilizing the surgical and IUE tools before start, and deposit plasmid DNA gently.

#### Problem 9

Only some of the pups have dual IUE, while others have a single IUE.

#### Potential Solution

This is likely because one of the IUEs was unsuccessful, or the second IUE was performed out of sequence and did not target the electroporated embryos. Some embryos could die between the first and second IUE. A dying embryo has an opaque white-yellowish color prior to being fully resorbed. Be mindful to count all the embryos carefully during the first and second IUEs, and note which embryos are no longer healthy. Skip unhealthy or dying embryos, even if they were part of the first IUE. When skipping those dying embryos, make sure to remain in sequence with the previous IUE pattern so that all healthy embryos that received the first IUE (E13.3) also receive the second IUE (E15.5).

## Data Availability

The original contributions presented in the study are included in the article/Supplementary Material, further inquiries can be directed to the corresponding author.
